# Structure and RNA template requirements of *Arabidopsis* RNA-DEPENDENT RNA POLYMERASE 2

**DOI:** 10.1073/pnas.2115899118

**Published:** 2021-12-13

**Authors:** Akihito Fukudome, Jasleen Singh, Vibhor Mishra, Eswar Reddem, Francisco Martinez-Marquez, Sabine Wenzel, Rui Yan, Momoko Shiozaki, Zhiheng Yu, Joseph Che-Yen Wang, Yuichiro Takagi, Craig S. Pikaard

**Affiliations:** ^a^HHMI, Indiana University, Bloomington, IN 47405;; ^b^Department of Biology, Indiana University, Bloomington, IN 47405;; ^c^Department of Molecular and Cellular Biochemistry, Indiana University, Bloomington, IN 47405;; ^d^Department of Biochemistry and Molecular Biology, Indiana University School of Medicine, Indianapolis, IN 47405;; ^e^CryoEM Facility, Howard Hughes Medical Institute, Janelia Research Campus, Ashburn, VA 20147;; ^f^Indiana University Electron Microscopy Center, Indiana University, Bloomington, IN 47405

**Keywords:** RDR2, RNA silencing, siRNA biogenesis, Pol IV–RDR2 coupling, polymerase backtracking

## Abstract

RNA-dependent RNA polymerases play essential roles in RNA-mediated gene silencing in eukaryotes. In *Arabidopsis*, RNA-DEPENDENT RNA POLYMERASE 2 (RDR2) physically interacts with DNA-dependent NUCLEAR RNA POLYMERASE IV (Pol IV) and their activities are tightly coupled, with Pol IV transcriptional arrest, induced by the nontemplate DNA strand, somehow enabling RDR2 to engage Pol IV transcripts and generate double-stranded RNAs. The double-stranded RNAs are then released from the Pol IV–RDR2 complex and diced into short-interfering RNAs that guide RNA-directed DNA methylation and silencing. Here we report the structure of full-length RDR2, at an overall resolution of 3.1 Å, determined by cryoelectron microscopy. The N-terminal region contains an RNA-recognition motif adjacent to a positively charged channel that leads to a catalytic center with striking structural homology to the catalytic centers of multisubunit DNA-dependent RNA polymerases. We show that RDR2 initiates 1 to 2 nt internal to the 3′ ends of its templates and can transcribe the RNA of an RNA/DNA hybrid, provided that 9 or more nucleotides are unpaired at the RNA’s 3′ end. Using a nucleic acid configuration that mimics the arrangement of RNA and DNA strands upon Pol IV transcriptional arrest, we show that displacement of the RNA 3′ end occurs as the DNA template and nontemplate strands reanneal, enabling RDR2 transcription. These results suggest a model in which Pol IV arrest and backtracking displaces the RNA 3′ end as the DNA strands reanneal, allowing RDR2 to engage the RNA and synthesize the complementary strand.

## Significance

RDR2 is critical for siRNA-directed DNA methylation in *Arabidopsis*, functioning in physical association with DNA-dependent Pol IV to synthesize the second strands of double-stranded siRNA precursors. Base-pairing between the DNA template strand transcribed by Pol IV and the nontemplate DNA strand is needed to induce Pol IV arrest and Pol IV/RDR2 transcriptional coupling, but how this occurs is unknown. We report the structure of RDR2 and experimental evidence for how RDR2 engages its RNA templates and initiates transcription. RDR2 engages the ends of RNAs displaced from RNA/DNA hybrids, suggesting a model in which Pol IV arrest and backtracking, accompanied by DNA strand reannealing, extrudes the 3′ end of the Pol IV transcript, allowing RNA engagement and second-strand synthesis by RDR2.

RNA-dependent RNA polymerases (RDRs) are encoded within the genomes of many eukaryotes and function in gene silencing by converting single-stranded RNAs (ssRNAs) into double-stranded (ds) precursors of short-interfering RNAs (siRNAs) (1, 2). The siRNAs associate with Argonaute family proteins and base pair with target locus RNAs to interfere with their translation or bring about transcriptional silencing at the corresponding chromosomal loci (3–5).

In plants, transcriptional silencing via siRNA-directed DNA methylation requires RNA-DEPENDENT RNA POLYMERASE 2 (RDR2), which associates with DNA-dependent NUCLEAR RNA POLYMERASE IV (Pol IV) (6, 7) via direct physical interactions that bring their active sites into proximity (8). The coupled transcription reactions of the Pol IV–RDR2 complex yield short dsRNAs of ∼30 bp (9, 10) that are then diced into 24- or 23-nt siRNAs by DICER-LIKE3 (DCL3) (9, 11–13). These siRNAs are then loaded into an Argonaute protein, primarily AGO4 (12), and guide the resulting complexes to target loci via base-pairing interactions with noncoding transcripts synthesized by NUCLEAR RNA POLYMERASE V (Pol V) (14, 15). Subsequent recruitment of DNA methyltransferase DRM2 and histone-modifying enzymes alters the local chromatin environment, inhibiting promoter-dependent transcription by RNA polymerases (RNAPs) I, II, or III (11, 15–18). In this manner, thousands of loci, mostly encoding transposable elements, are transcriptionally silenced throughout the genome. Analogous RDR-dependent transcriptional silencing pathways repress transposable elements in fission yeast, *Neurospora*, and nematodes (19–23).

Important mechanistic insights into the Pol IV–RDR2 partnership have come from recapitulation of siRNA biogenesis in vitro (11). Although Pol IV and RDR2 stably associate, they do not produce dsRNA when provided with only a ssDNA oligonucleotide template. Instead, single-stranded Pol IV transcripts are generated, which remain associated with the template as persistent RNA/DNA hybrids (6, 11). However, if a nontemplate DNA oligonucleotide is annealed to the template DNA strand, Pol IV arrests after transcribing ∼12 to 16 nt into the base-paired DNA region and RDR2 transcription now occurs, yielding dsRNAs that are released from the Pol IV–RDR2 complex (11). How base-pairing between the DNA template and nontemplate strands brings about Pol IV arrest and RDR2 coupling remains unclear.

Structural information for eukaryotic cellular RDRs is currently limited to crystal structures for two fungal QDE-1 (Quelling Defective-1) enzymes. One is a partial structure for the *Neurospora crassa* enzyme, missing amino acids 1 to 376, and the other is a partial structure for *Thermothielavioides terrestris* QDE-1 missing amino acids 1 to 363 (24, 25). We have determined the full-length structure of *Arabidopsis* RDR2 at 3.1 Å overall resolution by single-particle cryoelectron microscopy (cryo-EM). Striking structural similarities are apparent between the active centers of RDR2, QDE-1 enzymes, and multisubunit DNA-dependent RNA polymerases. The N-terminal region of RDR2 includes an RNA recognition motif (RRM) proposed to help define the RNA template path. Using oligonucleotides that recapitulate the configuration of the DNA template, DNA nontemplate, and RNA strands upon Pol IV arrest, we show that template–nontemplate DNA strand reannealing displaces the RNA 3′ end from the template DNA, making it available for RDR2 engagement. Because RNA strand displacement and extrusion also coincides with template–nontemplate DNA strand reannealing when multisubunit RNA polymerases undergo backtracking, we propose that Pol IV arrest and backtracking are the likely mechanisms by which Pol IV transcripts are channeled to RDR2.

## Results

### Expression and Single-Particle cryo-EM of Recombinant RDR2.

**Fig. 1.**
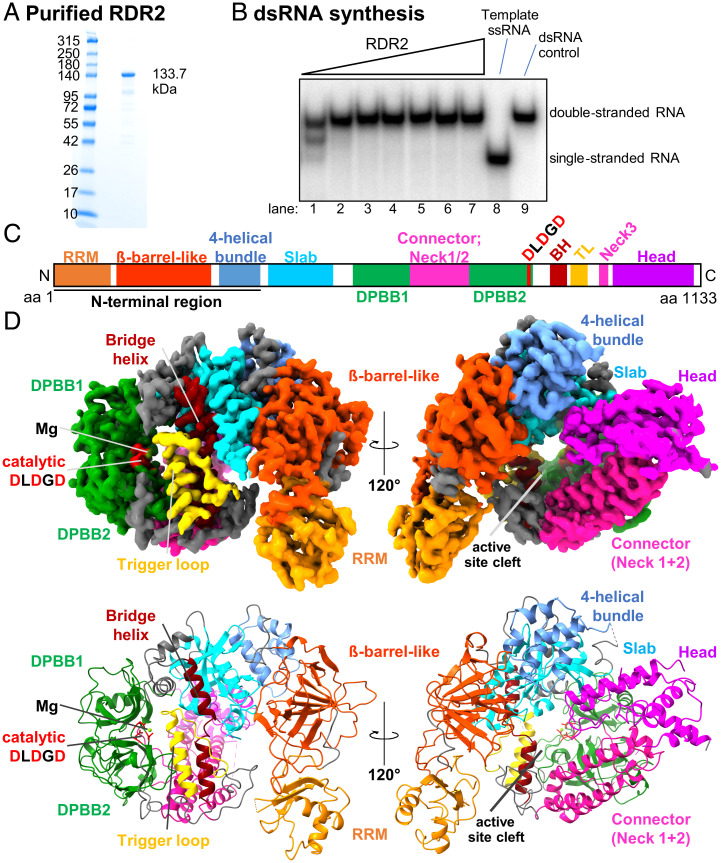
Structure of RDR2. (*A*) SDS/PAGE gel stained with Coomassie brilliant blue showing recombinant RDR2 used in this study. (*B*) dsRNA synthesis by recombinant RDR2. RDR2 (0.11 to 3.6 μM) was incubated with ssRNA and NTPs. Products were resolved by 15% native PAGE. (*C*) Domains of RDR2: RRM shown in orange; β-barrel–like (dark orange); four-helical bundle (cornflower blue); slab (blue); DPBB (green); connector/neck helices (magenta); BH: bridge helix (brown); TL: trigger-loop (yellow); and head (purple). The catalytic site (Metal A site), DLDGD is shown in red. (*D*) Ribbon diagram of RDR2 structure. Domains are colored as in *C*.

Full-length RDR2 expressed in insect cells using a baculovirus vector (Fig. 1 *A* and *B*) was subjected to single-particle imaging using a Titan Krios instrument equipped with a Gatan K3 detector. Images were collected with no tilt of the specimen grid or with 30° tilt to circumvent preferred particle orientation biases. Following two-dimensional (2D) classification, particle images from both collection methods were combined and used to generate an initial model. Successive rounds of three-dimensional (3D) classification, 3D refinement, contrast transfer function (CTF) refinement, and particle polishing were then conducted using RELION 3.1 (26–28), resulting in a map (Map 1) with 3.1 Å overall resolution (*SI Appendix*, Figs. S1–S4). Processing of the same datasets using cryoSPARC v3.2.0 (29) resulted in a very similar map (Map 2) with 3.57 Å overall resolution (*SI Appendix*, Figs. S5–S7; see *SI Appendix*, Fig. S7 *C*–*F* for comparisons of Map 1 and Map 2 views of the complete structure). The higher-resolution RELION map was then used for model building, employing Buccaneer (30) and manual steps. I-TASSER (31) was used to aid model building for the amino terminus of RDR2 (amino acids 1 to 100) due to relatively low-quality density for this region. Details of the model building process are provided as *SI Appendix*, *Detailed Methods*.

### Overall Structure of RDR2.

The N-terminal region (amino acids 1 to 360) of RDR2 begins with an N-terminal domain (amino acids 1 to 100) that includes an RRM followed by a β-barrel–like domain and a four-helical bundle domain (Fig. 1 *C* and *D*). Following the N-terminal region is an active site cleft composed of multiple domains termed slab, DPBB1, connector helices neck 1 and neck 2, DPBB2, bridge helix, trigger loop, connector helix neck 3, and head, respectively (Fig. 1*C*) (25, 32–34). DPBB (double-ψ β-barrel) domains 1 and 2 are linked by a connector domain, as in *Neurospora* QDE-1 and phi14:2 phage polymerase (34), which extends from DPBB1 and includes a short α-helix and part of the Neck 1 helix, resembling the “anchor” element of the yeast Pol II subunit, RPB2, that leads into the Pol II clamp domain (*SI Appendix*, Fig. S8). The three highly conserved aspartates (Asp triad) required for phosphodiester bond catalysis are located within a loop of DPBB2.

Following DPBB2, a loop passes across a potential secondary channel and connects to the bridge helix domain, followed by the trigger loop. Neck helix 3, part of which was not modeled due to flexibility in the loop, connects to the C-terminal head domain, composed of two long α-helices and a loop (again, not fully modeled due to flexibility) that protrudes into the cleft.

### Structural Conservation within RDR2 and Multisubunit RNAP Catalytic Centers.

**Fig. 2.**
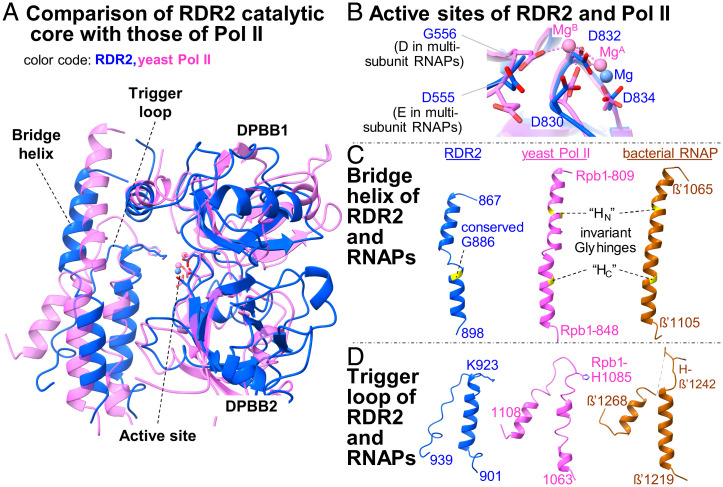
Structural comparisons of RDR2 with multisubunit RNAPs. Superimposition of RDR2 catalytic core structural elements with those of *S. cerevisiae* Pol II (PDB ID code 2E2H) and bacterial *T. thermophilus* RNAP (PDB ID code 4Q4Z). RDR2 colored in blue, yeast Pol II in magenta, and bacterial RNAP in brown. (*A*–*D*) Comparison of the active site (*B*), the bridge helix (*C*), and the trigger loop (*D*). For each domain, corresponding amino acid regions are shown from the same models superimposed (*A*), following the same color code. Invariant glycines in the bridge helix are highlighted in yellow, and two molecular hinges (HN and HC) in multisubunit RNAPs are indicated in broken lines. Invariant histidines in the trigger loop of multisubunit RNAPs and corresponding lysine resides in RDR2 are indicated.

A superimposition of the RDR2 active site loop (amino acids 828 to 836) of DPBB2 onto the corresponding structure of *Saccharomyces cerevisiae* Pol II is shown in Fig. 2*A*. The RDR2 and yeast Pol II active site loops share similarity to almost the same degree (RMSD = 0.649 Å) as the similarity between bacterial RNAP (*Thermus thermophilus*) and yeast Pol II (RMSD = 0.431 Å). Key domains of the catalytic core, including the two DPBBs, bridge helix, trigger loop, and Asp-triad align nearly perfectly (Fig. 2*B*). The Asp triad of multisubunit RNAPs corresponds to the Metal A site, which stably coordinates a magnesium ion at the site of catalysis (32). Extra density at the position of the three Asp residues is seen in the RDR2 EM map, likely corresponding to a Metal A Mg^2+^ ion (Fig. 2*B*). In structures for multisubunit RNAPs engaged in transcription, a second Mg^2+^ ion, Metal B, is observed in association with each incoming nucleotide triphosphate (35, 36). No Metal B density is observed in the RDR2 structure, but this is expected given that the RDR2 particles imaged by cryo-EM were not engaged in transcription. However, it will be interesting to learn in future studies how RDR2 and other RDRs coordinate Metal B ions. In multisubunit RNAPs, an invariant Glu-Asp motif coordinates Metal B. However, an Asp-Gly motif is found at the corresponding position in the catalytic centers of RDR2 and other RDRs, as shown in a multiple alignment of 5 RDR-γ clade (RDRγ) members and 11 α-clade (RDRα) members (*SI Appendix*, Figs. S9–S11) (2).

In multisubunit RNAPs, the bridge helix is a single α-helix within which two invariant glycines serve as molecular hinges (BH-H_N_ and BH-H_C_), providing conformational flexibility during translocation of the RNA and DNA chains (37–39). In RDR2, two α-helices connected by a short loop (amino acids 880 to 884) comprise the corresponding structure (Fig. 2*C*). A glycine (G886) conserved in nearly all cellular RDRs examined (*SI Appendix*, Fig. S10) is located at the junction between one of these helices and the intervening loop. The same structural arrangement is observed for the bridge helix of *Neurospora* QDE-1 (25). In *Arabidopsis* RDR6, a paralog of RDR2, mutation G921D, which changes the conserved glycine corresponding to RDR2 G886 to aspartic acid (G921D), results in a null mutant phenotype (40), providing genetic evidence for the importance of this glycine.

Beyond the bridge helix (amino acids 885 to 896) in the C-terminal direction is an α-helix (amino acids 906 to 922) followed by a loop (amino acids 923 to 939) that likely constitutes the trigger loop. In multisubunit RNAPs, the trigger loop is a flexible module that helps position substrate nucleotide triphosphates (NTPs) in the catalytic center during each nucleotide addition cycle (41, 42). The putative RDR2 trigger loop is shorter than those of multisubunit RNAPs and appears to resemble the “closed” conformation observed for substrate-bound multisubunit RNAP elongation complexes (Fig. 2*D*) (35, 36). The amino acid compositions of the bridge helices and trigger loops of RDR2 or QDE-1 differ from those of multisubunit RNAPs (Fig. 2*D* and *SI Appendix*, Fig. S11). In multisubunit RNAPs, an invariant histidine residue (His-β′1242 for *T. thermophilus* RNAP and His-1085 for yeast Rpb1) is critical for interaction with the β-phosphate of incoming NTPs. A lysine is present at the corresponding position in RDR2 (Lys-923), *Neurospora* QDE-1 (Lys-1119), and the crAss-like phage RNA polymerase, phi14:2 (Lys-1615) (34), suggesting that Lys-923 of RDR2 may be functionally equivalent to His-1085 of Pol II.

### The N-Terminal RRM Domain Has RNA Binding Activity.

**Fig. 3.**
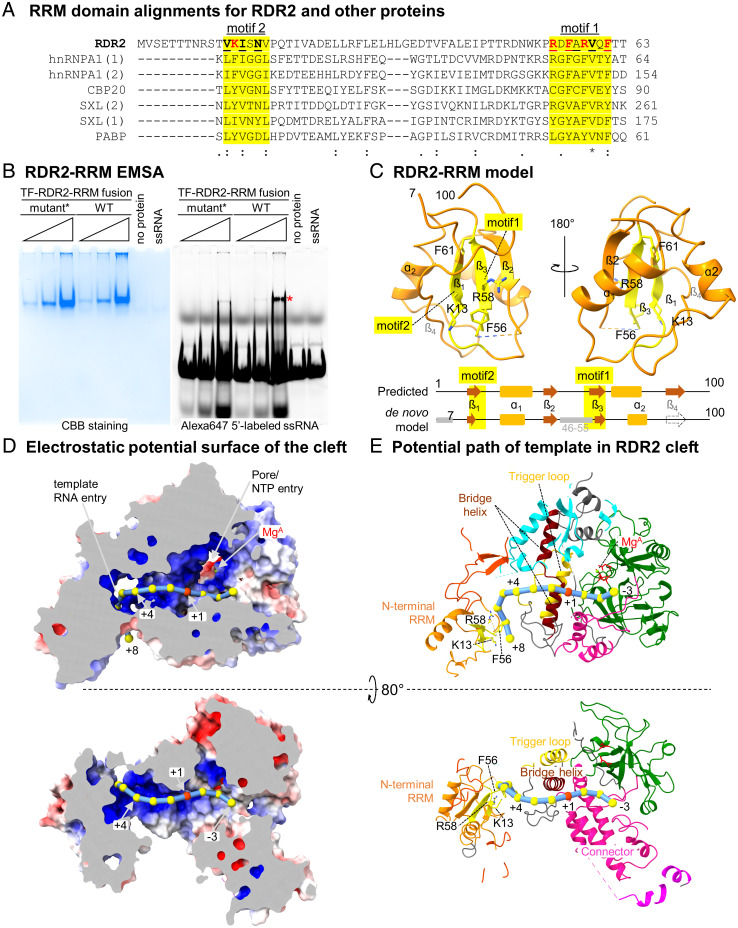
An N-terminal RRM domain influences the predicted path of template RNA. (*A*) RRM domain alignments for RDR2 (amino acids 1 to 63), human proteins: heterogeneous nuclear ribonucleoprotein A1 (hnRNPA), nuclear cap-binding protein subunit 2 (CBP) and polyA-binding protein 1 (PABP), and *Drosophila melanogaster* Sex lethal (SXL). Ribonucleoprotein 1 and 2 motifs (RNP1 and RNP2), having consensus sequences [RK]-G-[FY]-[GA]-[FY]-[ILV]-X-[FY] and [ILV]-[FY]-[ILV]-X-*N*-L, respectively, are shaded yellow. Basic or aromatic residues potentially interacting with RNA are shown in red. (*B*) EMSA of a recombinant TF-RDR2 (amino acids 1 to 100) fusion protein binding to a 37-nt RNA labeled with Alexa647. After acquiring the fluorescence image (*Right*), the gel was fixed and stained with Coomassie brilliant blue, CBB (*Left*). Wild-type and mutant forms of RDR2 were tested, with five amino acids of motifs 1 and 2 (shown in red in *A*) substituted by Ala in the mutant. An asterisk indicates the RNA–protein complex. (*C*) Structure of RDR2 (amino acids 1 to 100), with RRM domain motifs 1 and 2 colored yellow. The diagram at the bottom compares a computationally predicted secondary structure for the region to our structural model. (*D*) Electrostatic potential surface of RDR2 calculated using Adaptive Poisson–Boltzmann Solver (APBS) in Pymol with negative, neutral, and positive charges shown in red, white, and blue, respectively. RNA is modeled as beads on a string with the red bead indicating the position where complementary strand synthesis would begin (+1). Mg^2+^ and likely NTP entry pore positions are indicated. (*E*) Ribbon diagram views of the surface models shown in *D*. Domains are colored as in Fig. 1*D*. Additional views are shown in *SI Appendix*, Fig. S12.

RDR2 amino acids 7 to 95 are predicted to form a β-α-β-β-α-β–type RRM domain based on Genome3D analysis (43). Alignment to other RRM domain-containing proteins reveals similarities to the weak consensus sequences deduced for the so-called ribonucleoprotein motifs, RNP1 (amino acids 54 to 61) and RNP2 (amino acids 12 to 17) (Fig. 3*A*) (44). Consistent with these analyses, the structural model for the RDR2 N-terminal domain fits a β-α-β-β-α-β RRM fold, with the RNP1 and RNP2 motifs residing in the β3 and β1 elements but with the β4 element not apparent (Fig. 3*C*).

To test whether RDR2’s N-terminal domain (amino acids 1 to 100, hereafter referred to as RDR2-RRM) displays RNA-binding activity, we expressed in *Escherichia coli* RDR2-RRM fused, in-frame, to trigger-factor (TF). A fusion with a mutant form of RDR2-RRM^mut^, having five aromatic or positively charged amino acids of the RNP1 or RNP2 motifs changed to alanine, was also generated. Electrophoretic mobility shift assays (EMSA) demonstrated that TF-RDR2-RRM exhibits ssRNA binding that is substantially impaired in TF-RDR2-RRM^mut^, consistent with RNA binding activity (Fig. 3*B*).

### Prediction of the RNA Template Path.

A structure for RDR2 engaged with RNA has not yet been achieved, but structural and functional data suggest a potential path for the template RNA within RDR2. Examination of the electrostatic surface potential shows a positively charged surface, starting with the RDR2-RRM and continuing along the cleft and into the active site (Fig. 3*D*). If an RNA template is modeled along the positively charged surface, with the transcription initiation position at the Metal A site defined as nucleotide position +1, the RNA path is relatively straight from +1 to +5, but then changes trajectory to interact with the surface of the RRM that helps form the putative RNA entry channel (Fig. 3 *D* and *E*). In the opposite direction, the opening between the two DPBB domains and the connector domain is the presumed dsRNA exit channel (see “back” view, *SI Appendix*, Fig. S12).

### RDR2 Engages ssRNAs Longer than 7 nt and Initiates Internal to Their 3′ Ends.

**Fig. 4.**
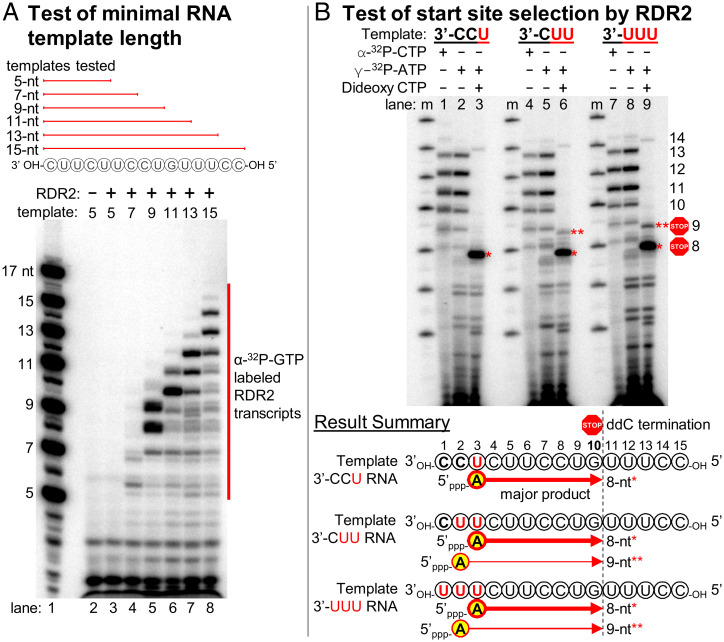
RDR2 initiates internal to its RNA templates. (*A*) Test of minimal RNA template length required for RDR2 transcription. ssRNAs of 5, 7, 9, 11, 13, or 15 nt were tested, with transcripts labeled by incorporation of α-[^32^P]-GTP. In lane 1, RNAs 5′ end-labeled using γ-[^32^P]ATP serve as size markers. (*B*) Test of start site selection by RDR2. Using 15-nt ssRNA templates that have 3′-CCU, 3′-CUU, and 3′-UUU at their 3′ ends, RDR2 transcription was carried out in the presence of α-[^32^P]-CTP, γ-[^32^P]ATP or γ-[^32^P]-ATP plus 2’,3′-dideoxy CTP (ddC).

The distance between the RNA-binding surface of RDR2-RRM and the catalytic site is 41 Å, which correspond to ∼7 nt of ssRNA, assuming a nucleotide spacing of ∼6 Å. Our model thus predicts that an RNA of ∼7 nt or longer would be needed to serve as a template for RDR2 (Fig. 3*E*). To test this prediction, transcription reactions were conducted using ssRNAs that varied in length from 5 nt to 15 nt. No ^32^P-labeled RNA transcripts were detected using the 5-nt template (Fig. 4 *A*, lane 3) and weak transcription occurred using the 7-nt template, but robust transcription occurred using templates of 9 nt or longer (Fig. 4 *A*, lanes 4 to 8).

Note that the most abundant transcripts in Fig. 4*A* are consistently 1 to 2 nt shorter than the templates (*SI Appendix*, Fig. S13), suggesting that transcription may initiate internal to the 3′ ends of template RNAs. To test this hypothesis, we designed three 15-nt RNA templates that differ in the 3 nt present at their 3′ ends: either 3′-CCU, 3′-CUU, or 3′-UUU (Fig. 4*B*). We then conducted RDR2 transcription reactions in which nascent transcripts were either body-labeled by incorporation of α-[^32^P]-CTP (Fig. 4 *B*, lanes 1, 4, and 7) or end-labeled by using γ-[^32^P]ATP as the initiating nucleotide (Fig. 4 *B*, lanes 2, 5, and 8). We also generated RNAs that initiated with γ-[^32^P]ATP but were then terminated at a fixed position complementary to the guanosine at template position 10 upon incorporation of the chain terminator, 2′,3′-dideoxy CTP (ddC) (Fig. 4 *B*, lanes 3, 6, and 9).

Labeling with α-[^32^P]-CTP or γ-[^32^P]ATP yielded similar transcription products for all three 15-nt templates, with transcripts of 8 to 13 nt being most abundant (compare lanes 1, 2, 4, 5, 7, and 8 in Fig. 4). Products of 14 nt were also detected, at much lower levels, but full-length 15-nt transcripts were undetectable or observed in only trace amounts. In the presence of ddC (Fig. 4 *B*, lanes 3, 6, and 9), a major labeled band of 8 nt was detected for all three templates, and a less abundant 9-nt band was observed for the 3′-CUU and 3′-UUU templates. Because only the 5′ terminal nucleotide retains the labeled γ-phosphate of γ-[^32^P]ATP, the production of an 8-nt transcript that terminates at template position 10 indicates that transcription initiated with an adenosine complementary to the uridine at template position 3 (see summary diagrams of Fig. 4*B*). Likewise, 9-nt transcripts initiated at template position 2. Collectively, these results show that RDR2 initiates at positions complementary to the second or third nucleotides internal to the template.

### RDR2 Can Transcribe the RNA of an RNA/DNA Hybrid if the 3′ End Is Accessible.

**Fig. 5.**
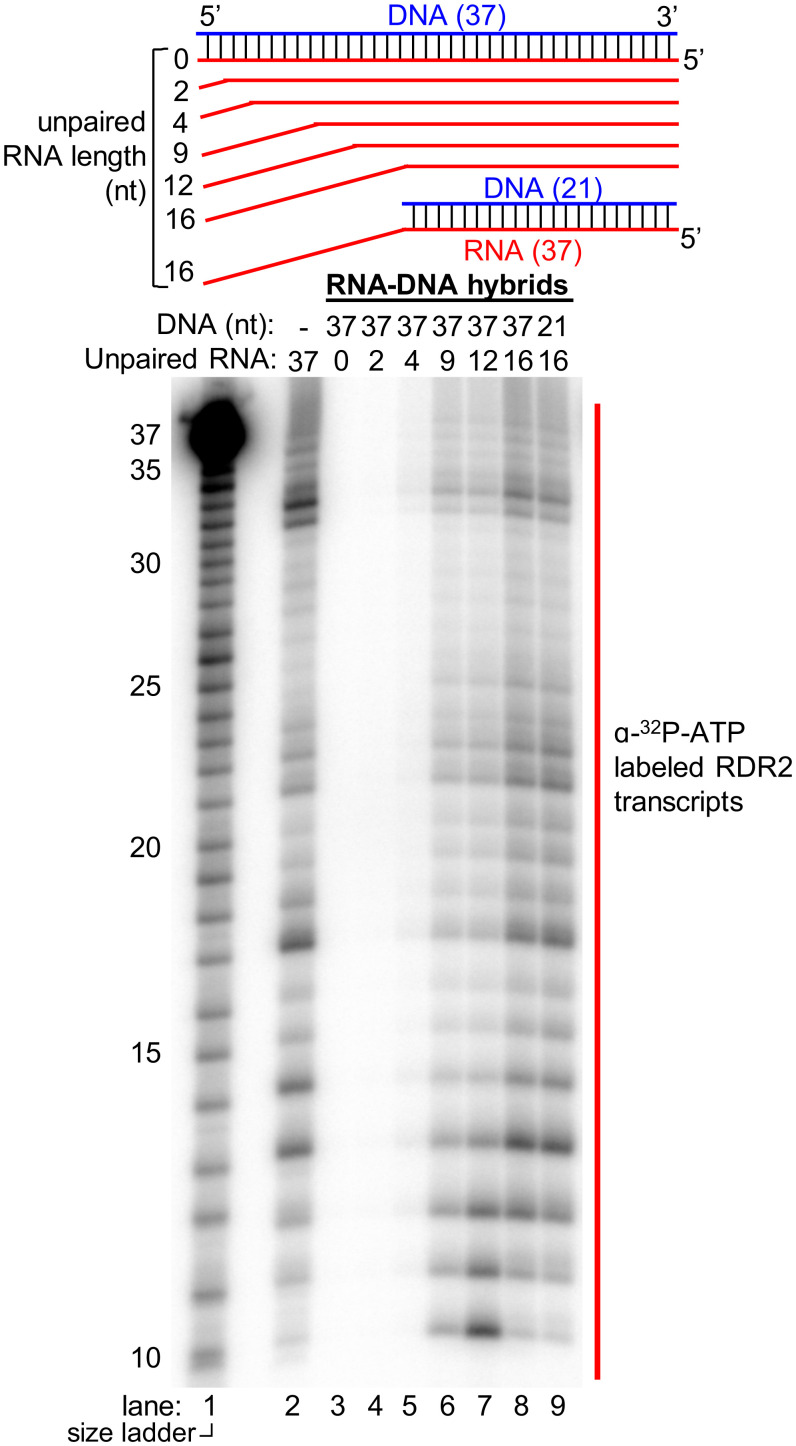
Test of RNA template engagement in the context of an RNA/DNA hybrid. As depicted in the diagram, 37-nt RNAs were hybridized to a 37-nt DNA strand to form hybrids with 0, 2, 4, 9, 12, or 16 nt of unpaired RNA at the 3′ end, then tested as templates for RDR2 transcription (lanes 3 to 8). A 21-nt DNA oligo was also used to generate a hybrid with 16 nt of unpaired RNA (lane 9). An RNA-only control (no DNA) was tested in lane 2 (same RNA as in lane 3). RNA transcripts were labeled by incorporation of α-[^32^P]ATP. Partially hydrolyzed 5′ end-labeled 37-nt RNA was used as a size ladder in lane 1.

How RDR2 engages Pol IV transcripts to generate dsRNAs is unknown. In vitro, Pol IV transcription of DNA templates in the absence of RDR2 yields persistent RNA/DNA hybrids, not free ssRNAs (11). To determine if RDR2 can access the RNA strand of RNA/DNA hybrids, we first tested a 37-nt RNA fully hybridized to a complementary 37-nt DNA. RDR2 fails to generate transcripts from this hybrid template (Fig. 5, lanes 2 and 3). However, if an unpaired, single-stranded region of 9 nt or longer is present at the 3′ end of the RNA, and the rest is base-paired with DNA, RDR2 can transcribe the RNA (Fig. 5, lanes 4 to 8). The unpaired portion of the DNA strand is dispensable for RDR2 engagement of the RNA 3′ end, as shown by the fact that it can be deleted without affecting RDR2 transcription of the remaining RNA/DNA hybrid (Fig. 5, lane 9; see bottom-most template diagram). The need for 9 or more unpaired nucleotides at the 3′ end of the RNA template is consistent with the minimal length of ssRNA needed for transcription (Fig. 4*A*) and is equivalent to the distance from the RRM domain to the active site (∼7 nt) plus the 2 extra nucleotides needed for initiation to begin at the third position internal to the template (Fig. 4*B*).

### Nontemplate–Template DNA Strand Reannealing Displaces the RNA Strand of RNA/DNA Hybrids to Enable RDR2 Transcription.

**Fig. 6.**
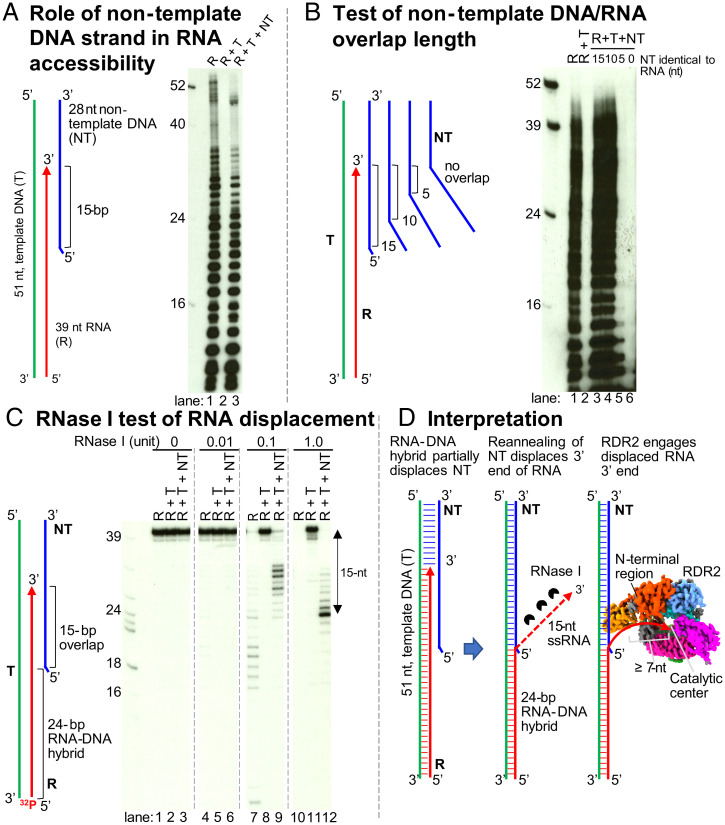
Role of nontemplate DNA in RDR2 engagement of RNAs in the context of RNA/DNA hybrids. (*A*) A nucleic acid configuration that mimics the situation when Pol IV arrests 15 nt into a downstream dsDNA region was prepared by annealing 39-nt RNA (R, red), 51-nt template DNA (T, green), and 28-nt nontemplate DNA strands (NT, green). RDR2 transcription was tested using R only (lane 1), R+T (lane 2), and R+T+NT combinations (lane 3), with transcripts labeled by incorporation of α-[^32^P]ATP. (*B*) Test of nontemplate DNA/RNA overlap length. Same assay as in *A*, but varying the NT strand to have 10-, 5-, or 0-nt of sequence identity (overlap) with the RNA strand. (*C*) RNase I test of RNA displacement. 5′ end-labeled R strands in R only, R+T, or R+T+NT combinations were examined at four RNase I concentrations. (*D*) Graphic interpretation of the results in *A*–*C*.

Coupling of Pol IV transcription and RDR2 transcription requires Pol IV transcriptional arrest within a dsDNA region (11). Previous assays of Singh et al. used a 51-nt DNA oligonucleotide template annealed, at its 5′ end, to a 28-nt nontemplate DNA strand, forming a 27-bp double-stranded region with a 1-nt unpaired flap at the nontemplate strand’s 5′ end (see diagram of Fig. 6*A*). Pol IV transcription was then initiated using an RNA primer hybridized to the template’s 3′ end, thus ensuring that resulting Pol IV transcripts have a defined 5′ end. Upon reaching the double-stranded portion of the template, Pol IV can only proceed an additional 12 to 16 nt before arresting or terminating, an event coupled to RDR2 engagement of the Pol IV transcript and synthesis of the complementary strand (11). Using the same 51-nt DNA template and 28-nt nontemplate DNA strands used by Singh et al., and a 39-nt RNA corresponding to a Pol IV nascent transcript that extended 15 nt into the dsDNA region, we examined the ability of RDR2 to access the RNA and convert it into dsRNA (Fig. 6*A*). When provided with the RNA alone, RDR2 generates abundant transcripts, forming a ladder of bands (Fig. 6 *A*, lane 1). Some of these bands are longer than full-length due to folding of the RNA into an asymmetric stem-loop and further elongation of the 3′ end using the single-stranded stem as a template. If the RNA is fully hybridized to the template DNA strand no RDR2 transcription occurs (Fig. 6 *A*, lane 2). However, if the nontemplate DNA strand is included in the annealing/hybridizing reactions, along with the DNA template and RNA strands, RDR2 is now able to access and transcribe the RNA strand (Fig. 6 *A*, lane 3).

We next tested the length of overlap needed between the nontemplate DNA strand and RNA strand in order for RDR2 to transcribe the RNA (Fig. 6*B*). As in Fig. 6*A*, controls (lanes 1 and 2) show that RDR2 can transcribe free RNA but not RNA fully hybridized to template strand DNA. Inclusion of a nontemplate DNA strand with 15 or 10 nt of identity to the 3′ end of the RNA enabled RDR2 transcription of the RNA strand (Fig. 6 *B*, lanes 3 and 4), whereas nontemplate strands with only 5 or 0 nt of overlap did not (Fig. 6 *B*, lanes 5 and 6).

The results of Fig. 6 *A* and *B* suggest that the nontemplate DNA strand competes with the RNA strand for base pairing to the template DNA strand, resulting in displacement of the RNA’s 3′ end, thereby enabling RDR2 to engage and transcribe the RNA. To test this hypothesis, we examined the RNase I sensitivity of the RNA strand alone or in the presence of the template and nontemplate DNA strands (Fig. 6*C*). For this experiment, the 39-nt RNA was labeled with ^32^P at its 5′ end. In the absence of complementary DNA, the RNA strand was readily digested by RNase I (Fig. 6 *C*, lanes 7 and 10) but when hybridized to the DNA template strand, the RNA was RNase-resistant (Fig. 6 *C*, lanes 5, 8, and 11). Inclusion of the nontemplate strand, along with the RNA and DNA template strands, resulted in RNase I trimming of the RNA, causing the accumulation of a prominent 24-nt product (Fig. 6 *C*, lanes 9 and 12). This trimming of 15 nt corresponds to the 15 nt of overlap between the RNA and nontemplate DNA strands, consistent with the competitive base-pairing hypothesis (Fig. 6*D*).

## Discussion

Cellular RDRs have been classified into three major lineages—RDRα, RDRβ, and RDRγ–with RDRα enzymes possessing the largest number of conserved motifs (2). *Arabidopsis* RDR2 belongs to the RDRα clade, thus we expect its structure to be informative for studies of other α-subfamily members involved in gene silencing, including *Arabidopsis* RDR1 and RDR6, *N. crassa* SAD-1, *Schizzosaccharomyces pombe* Rdp1, and *Caenorhabditis elegans* EGO-1/RRF1/RRF3. Fungal QDE-1 proteins represent the RDRγ clade. The catalytic cores of RDR2 and QDE-1 are similar. However, an intriguing difference between the enzymes is that RDR2 behaves as a monomer during purification (8) and upon imaging by cryo-EM, whereas the QDE-1 proteins were crystalized as homodimers. For QDE-1 enzymes, a “two-stroke motor” mechanism has been proposed, in which one dimer subunit becomes active (closed) upon RNA binding, while the other subunit becomes inactive (open) (24, 25). A possibility is that the sequential reactions of Pol IV and RDR2 may constitute an analogous two-stroke scenario.

### Genetic Results Relevant to the RDR2 Structure.

There is a considerable body of genetic evidence relevant to the RDR2 structure. The Asp-triad loop, amino acids 830-DLDGD-834, is known to be critical for catalytic activity, as shown by the loss of activity upon substituting three alanines for DGD at positions 832 to 834 or upon substituting glycine for aspartate at position 834 (8, 45). Moreover, a genetic screen recovered G833E as an RDR2 loss-of-function mutant (46).

In RDR6, a paralog of RDR2 (*SI Appendix*, Fig. S9*A*), mutations at numerous amino acid positions conserved in RDR2 disrupt posttranscriptional gene silencing, including: P611L within the DPBB1 domain; G825E, D826N, and S860F within the DPBB2 domain; G866E within the ASP-triad loop; G921D within the bridge helix; and E429K and E453K within the slab domain (40, 47–49) (*SI Appendix*, Fig. S14*A*).

The RDR2 head domain is composed of five α-helices. A nonsense mutation, W1083* that truncates the last 51 residues of the C-terminal head domain, was recovered as a silencing defective mutant (50). In *Zea mays*, a Mu transposon insertion at amino acid position 962, within the neck 3 helix leading to the head domain of the RDR2 ortholog, MOP1 resulted in the loss of MOP1 function (51). In RDR6, multiple nonsense mutations within the head domain disrupt silencing, including Q1145*, W1160*, Q1055* (48, 52), and W1039* (53) (*SI Appendix*, Fig. S14*B*). Collectively, these mutations provide genetic evidence for the importance of the head domain, yet the function of the domain remains unclear. In the RDR2 structure, the tip of the head domain faces toward the cleft (Fig. 1*D*). Importantly, our 3D reconstruction lacked density corresponding to the tip of the head (amino acids 1029 to 1045) but 3D variable analysis by cryoSPARC (54) captured the extension of the tip of the head pushing toward the cleft (Movie S1). We speculate that the tip of the head may interact with incoming template RNA, helping guide the RNA to the active site.

Functions for the β-barrel–like domain, four-helical bundle, and slab and neck domains remain unclear. We note that the slab domain of RDR2 contains structural features resembling the fork loops and link domain helix of yeast Pol II. Specifically, when superimposed, the RDR2 slab (amino acids 470 to 488) corresponds well with fork loop 3 of yeast Rpb2 (amino acids 521 to 541), whereas RDR2 amino acids 511 to 521 form an α-helix, which may correspond to the Rpb2 link domain helix (amino acids 757 to 776) (*SI Appendix*, Fig. S15). The fork domain composed of loop 1 to 3 (amino acids 466 to 546) of Rpb2 contacts the RNA/DNA hybrid, the template strand, and downstream DNA (36, 55, 56), whereas amino acids of the link domain interact with NTPs (39). Thus, it is possible that the slab domain of RDR2 may interact with template RNA and NTPs.

### A Potential Structural Basis for RDR2 Initiation Internal to Template 3′ Ends.

For multisubunit RNAPs, the initiating NTP (iNTP) is stabilized by interactions with conserved positively charged or polar amino acids (e.g., *T. thermophilus* β-subunit amino acids Gln-567, Lys-838, Lys-846, and His-999; or corresponding *S. cerevisiae* Pol II Rpb2 subunits Gln-776, Lys-979, Lys-987, and His-1097) by base-stacking interactions with the template base at position −1 (1 nt upstream of the initiation site, +1) and by water-mediated interactions between a phosphate group of the iNTP and template bases at the −1 and −2 positions (*SI Appendix*, Fig. S16) (57). We questioned whether analogous interactions might explain RDR2’s initiation 2 to 3 nt internal to its templates. In the RDR2 structure, positively charged or polar residues Lys-589 (equivalent to *T. thermophilus* Lys-846) and Gln-582 (equivalent to *T. thermophilus* Lys-838) are present in the equivalent iNTP α-phosphate interacting positions (*SI Appendix*, Fig. S16) and are invariably conserved among the RDRs examined. However, potential iNTP γ-phosphate interacting amino acids are less conserved in RDR2 and other RDRs (*SI Appendix*, Fig. S10). In the position equivalent to *T. thermophilus* His-999, RDR2 has Arg-622, whereas other RDRs have Lys, Asn, or other amino acids (*SI Appendix*, Figs. S10 and S16). Polar amino acids are conserved at RDR positions corresponding to RDR2 Ser-525 and may correspond to *T. thermophilus* Gln-567. These structural comparisons suggest that initiation site selection by RDR2 may have some mechanistic similarities to initiation site selection in multisubunit RNAPs, a possibility worthy of further structural investigation.

Importantly, the fact that RDR2 initiates internal to the 3′ ends of its RNA templates has biological significance, because it causes the resulting dsRNA to have a 1- or 2-nt 3′ overhang. In other studies (58), we have found that dsRNAs with 1- or 2-nt 3′ overhangs are the preferred substrates for DCL3. Thus, internal initiation by RDR2 plays a direct role in siRNA biogenesis.

### A Model for RDR2 Engagement of Backtracked Pol IV Transcripts.

The transcription reactions of Pol IV and RDR2 are tightly coupled, with Pol IV arrest and RDR2 initiation of second-strand RNA synthesis being linked processes dependent on base-pairing interactions between the DNA template and DNA nontemplate strands (11). RDR2 can readily transcribe ssRNA templates. However, single-stranded Pol IV transcripts are not released by Pol IV for RDR2 to engage. Instead, in the absence of RDR2, or in the presence of RDR2 but absence of the nontemplate DNA strand, the Pol IV transcripts base pair with the template DNA to form persistent RNA/DNA hybrids that RDR2 cannot transcribe. However, if the nontemplate DNA strand is present, dsRNAs are now synthesized, and are released (11). Our present study shows that RDR2 can only engage the RNA of an RNA/DNA hybrid if ∼9 nt (or more) of the RNA’s 3′ end is unpaired from the DNA. Moreover, we’ve shown that template–nontemplate strand reannealing can displace the 3′ end of the RNA, thereby enabling RDR2 to engage and transcribe it into dsRNA (Figs. 5 and 6).

**Fig. 7.**
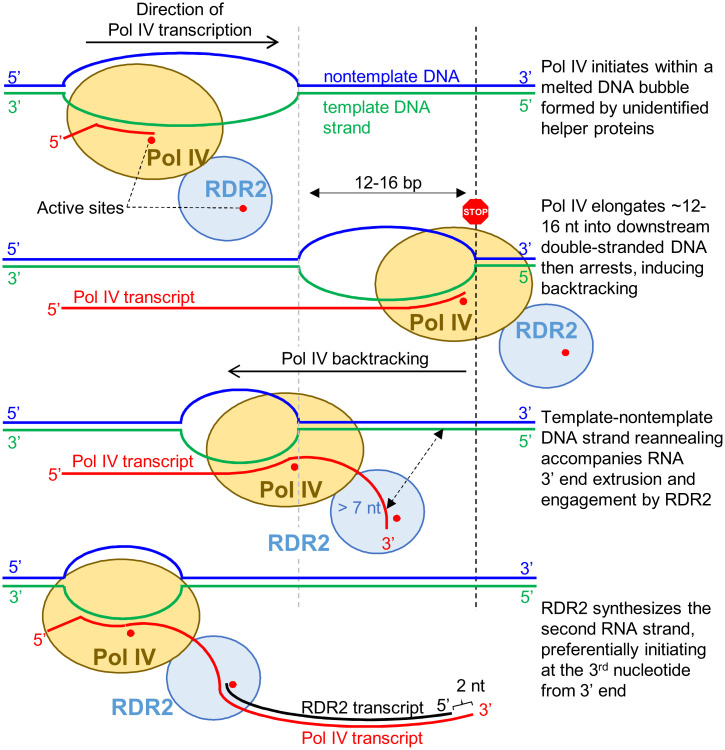
Backtracking model for Pol IV–RDR2 transcription coupling.

Importantly, multisubunit RNAPs in eukaryotes and bacteria have been shown to catalyze this very reaction, a phenomenon known as backtracking (59, 60). Upon encountering an obstacle to transcription elongation, such as a DNA lesion, polymerase arrest is followed by reverse translocation (backtracking) along the template DNA, with the nascent transcript’s 3′ end extruded through the so-called secondary channel as the template and nontemplate strands of DNA reanneal. Collectively, these considerations lead us to propose the model shown in Fig. 7. In this model, Pol IV initiates on one DNA strand within an initiation bubble but upon encountering dsDNA can elongate only ∼12 to 16 nt before arresting, possibly as a result of amino acid substitutions and deletions in the “rudder” and “zipper” loops implicated in transcription bubble perpetuation (11, 61, 62). Unable to go forward, we propose that Pol IV backtracking ensues, with reannealing of the nontemplate and the template DNA strands occurring as the RNA 3′ end is displaced and extruded (59). When the length of the extruded RNA is long enough (at least 9 nt), RDR2 engages the RNA and initiates second-strand synthesis, with Pol IV continuing to backtrack until the dsRNA is completed and released from the Pol IV–RDR2 complex. This model accounts for our biochemical results to date, including the role of the nontemplate DNA strand, the linkage between Pol IV arrest and RDR2 initiation, the extrusion of Pol IV transcript 3′ ends to make them accessible to RDR2, and the release of only double-stranded, not single-stranded, RNAs from the Pol IV–RDR2 complex. We envision that single-molecule studies may best allow the model to be tested by revealing the relative movements of the enzymes and nucleic acids during the coupled transcription reactions of Pol IV and RDR2.

## Materials and Methods

### Construction of a Baculovirus Vector for Expression of RDR2.

A baculovirus vector termed pSEP10 that allows for the expression of large proteins in insect cells was used to produce RDR2. Briefly, a synthetic RDR2 open reading frame was cloned into the BamHI and Hind III sites of pSEP10, resulting in the SEP tag being fused in-frame to the RDR2 open reading frame and a Twin-Strep tag at the C terminus. Details are provided in *SI Appendix*, *Detailed Methods*. Generation of baculovirus expressing RDR2 using the resulting pSEP10-RDR2 vector was performed as described previously (63).

### Expression and Purification of RDR2.

Expression of recombinant RDR2 was optimized using the TEQC method (63) in which a 200-mL culture of Hi5 cells (Expression Systems) was infected at an estimated multiplicity of infection of 4. After a 96-h incubation at 27 °C, cells were harvested, frozen in liquid nitrogen, and kept at −80 °C until use. Purification of recombinant RDR2 from lysed cell pellets was carried out by Strep-Tactin affinity purification followed by Hitrap Q column chromatography. Details are provided in *SI Appendix*, *Detailed Methods*. Hitrap Q fractions containing the recombinant RDR2 were concentrated using a spin column (100-kDa cutoff) to a final concentration of 4.8 mg/mL, as measured using a Bradford assay.

### Cryo-EM Sample Preparation and Data Collection.

Grid preparation, grid screening, and data collection were performed in the cryo-EM facility at the HHMI Janelia Research Campus. Datasets were collected with no stage tilt or with 30° of stage tilt using a Titan Krios microscope (Thermo Fisher) operated at 300 kV and equipped with a K3 camera (Gatan). Detailed methods for grid preparation and data collection are described in *SI Appendix*, *Detailed Methods*.

### Image Processing.

The map used to build the de novo atomic model was obtained by image processing using Relion 3.1 (27, 28, 64). The workflow and detailed procedures for each step are described in the *SI Appendix*, *Detailed Methods* and Figs. S1 and S2. Briefly, after beam-induced motion correction and CTF estimation, an initial 3D model at ∼4.3-Å resolution was generated from 2D-reference–picked particles, which was then used as a 3D-reference to select a subset of particles. Two-dimensional classifications, 3D classifications, iterative CTF refinement, Bayesian polishing, and metadata filtering of these selected particles led to the final map, “Map1” of ∼3.10 Å. Independent processing of the same dataset by cryoSPARC v3.2.0, which resulted in a nearly identical 3D reconstruction at ∼3.57 Å, “Map2”, is described in *SI Appendix*, *Detailed Methods* and Fig. S3. All figures for cryo-EM density maps, models and model surfaces were generated using Chimera v1.15 (65) or Chimera X software (66).

### Model Building.

Map1 was sharpened using the DeepEMhancer tool (67) of the COSMIC^2^ science gateway (68) (Map1) then used for model building. Model building for amino acid residues 61 to 1121 of Map1 was carried out de novo using the automated program Buccaneer (30) and Emap2sec (69) was used to assess secondary structure propensity in the EM map. Model building for residues 1 to 60 was aided by a computational model generated by I-TASSER (31). Detailed methods for the model building and refinement process are described in *SI Appendix*, *Detailed Methods*.

### RDR2 Tanscription Assays.

RDR2 transcription assays were conducted by mixing templates (RNA or RNA/DNA hybrids), NTPs supplemented with [^32^P]-labeled ATP, GTP or CTP, and RDR2. After incubating at room temperature to 27 °C for 1 to 2 h, the reactions were either treated by proteinase K or passed through PERFORMA spin columns (Edge Bio), precipitated, and resolved by 15% or 17% denaturing PAGE. The gels were then subjected to either phosphorimaging or autoradiography using X-ray films. In Fig. 1*B*, the reaction was analyzed by 15% native PAGE instead to detect dsRNA. Details are provided in the *SI Appendix*, *Detailed Methods*.

### Expression and Purification of Recombinant TF-RDR2-RRM Fusion Protein.

To produce recombinant the TF-RDR2-RRM fusion protein, the DNA sequence encoding RDR amino acids 1 to 100 (or a mutant version containing five alanine substitutions as described in Fig. 3), was codon-optimized for *E. coli*, synthesized by GenScript, and cloned into the pCold-TF vector (Takara) BamHI and HindIII site. The resulting construct was transformed into ArcticExpress competent cells (Agilent). Protein expression was induced by cold shock. The fusion protein was affinity purified using Ni-TNA agarose (Qiagen). Details are provided in *SI Appendix*, *Detailed Methods*.

### EMSA.

Recombinant TF-RDR2-RRM proteins (at 9, 3, or 1 μM) were incubated with a 37-nt ssRNA (final 2 μM), 5′ end-labeled with Alexa647, in 25 mM Hepes-KOH pH7.6, 50 mM NaCl, 2 mM MgCl_2_, and four units of RNase Inhibitor Murine (New England Biolabs, M0314). After 30 min at room temperature, reactions were subjected to 6% native PAGE and fluorescence imaging followed by staining with Coomassie brilliant blue.

## Supplementary Material

Supplementary File

Supplementary File

## Data Availability

Structural data have been deposited in PDB, https://www.rcsb.org/ (PDB ID codes 7ROZ and 7RQS) and the Electron Microscopy Data Bank, https://www.ebi.ac.uk/emdb/ (EMDB ID codes EMD-24610 and EMD-24635).
